# The ocean within planetary boundaries: complexity, tipping risks and pathways to a safe operating space

**DOI:** 10.1093/nsr/nwag630

**Published:** 2026-10-06

**Authors:** Minhan Dai, Jingfang Fan, Jürgen Kurths, Véronique Garçon

**Affiliations:** State Key Laboratory of Marine Environmental Science & College of Ocean and Earth Sciences, Xiamen University, China; Fujian Ocean Innovation Center, China; School of Systems Science/Institute of Non-equilibrium Systems, Beijing Normal University, China; Potsdam Institute for Climate Impact Research, Germany; Potsdam Institute for Climate Impact Research, Germany; Research Institute of Intelligent Complex Systems, Fudan University, China; Centre National de la Recherche Scientifique, Institut de Physique du Globe de Paris, France

The ocean is a central regulator of the Earth system. It absorbs and redistributes heat and anthropogenic carbon, sustains biodiversity and biogeochemical cycles, and supports human societies worldwide. Although ocean processes underpin several planetary boundaries (PBs), their representation within the framework remains uneven. Ocean acidification is the only boundary explicitly focused on the marine realm, while ocean circulation, deoxygenation and marine ecosystem functioning are insufficiently captured by existing control variables. A central challenge is therefore to represent these marine functions more adequately and link regional ecological change to Earth-system stability.

Expanding ocean uses, including high-seas fisheries, proposed deep-sea mining and ocean-based climate interventions, heighten the need to assess cumulative pressures and trade-offs. As the Agreement under the United Nations Convention on the Law of the Sea on the Conservation and Sustainable Use of Marine Biological Diversity of Areas beyond National Jurisdiction (BBNJ Agreement) moves into implementation, the need for a coherent scientific framework to inform precautionary ocean governance becomes increasingly pressing. Marine conservation also requires an understanding of how local protection interacts with wider environmental change. Stronger marine representation in PB assessments could help address these needs alongside regional and sector-specific approaches. This Special Topic, ‘Complex Marine Ecosystems and Planetary Boundaries’, brings together six contributions that explore these challenges through Earth-system science, marine biogeochemistry and complexity science.

Mathesius *et al.* [1] place the ocean in the broader Earth-system context, examining its roles in climate regulation, carbon storage and biosphere functioning, and identifying candidate marine processes and control variables for inclusion in the PB framework. Findlay and Feely [2] revisit the ocean acidification boundary in light of recent evidence indicating that it has already been transgressed. Their discussion of regional and depth-dependent heterogeneity, biological responses and the choice of control variables highlights a broader challenge: global averages can obscure conditions critical to marine organisms and ecosystem functioning. The accompanying Forum, chaired by Minhan Dai [3], examines gaps in the current framework and pathways toward better incorporating circulation, deoxygenation, marine ecosystem integrity and anthropogenic pressures into future assessments.

PBs and tipping points offer complementary perspectives. Boundaries identify precautionary limits to anthropogenic perturbation, whereas tipping analysis examines how systems may undergo abrupt or self-sustaining transitions. Crossing a boundary does not necessarily imply an immediate tipping event, but interactions among processes may amplify the risk of such transitions. Fang *et al.* [4] review tipping points and cascading transitions through the lens of nonlinear dynamics and complexity science, bringing together bifurcation-, noise- and rate-induced tipping, early-warning indicators, network approaches and emerging artificial intelligence methods. Meng and Chen [5] examine how interactions among tipping elements across physical, biogeochemical, ecological and socioeconomic domains can propagate risks through multilayer networks. Applied to the ocean, this perspective provides a basis for investigating potential cascades linking circulation changes, deoxygenation, ecosystem degradation and fisheries decline.

Zhan *et al.* [6] examine the Arabian Seas as a regional example of interacting climatic, biogeochemical and ecological pressures. Warming, changes in circulation and ventilation, deoxygenation, acidification and ecological reorganization illustrate how multiple pressures can combine to reshape marine systems. The region offers insight into mechanisms relevant to other tropical and marginal oceans, while underscoring the importance of regional context when interpreting planetary-scale risks.

Together, these contributions identify a common task: to represent the ocean’s regulating functions more adequately within PB assessments while accounting for regional heterogeneity and interactions among processes. Priorities include developing and testing marine control variables, determining how regional ecological thresholds relate to Earth-system risk, and evaluating how interacting pressures alter resilience and tipping potential. Sustained observations and mechanistic models across scales are essential to this effort. Incorporating tipping dynamics into these models could substantially improve assessments of abrupt transitions and cascading risks, although this remains a major scientific challenge. Digital twin technologies could support these efforts by integrating observations and models to explore future scenarios and assess the consequences of alternative interventions. Such advances would strengthen the scientific basis for assessing cumulative ocean pressures and guiding development, conservation and climate interventions within a safe operating space for humanity.
